# Neural frequency-tagging of syllables in naturalistic speech is sensitive to background noise and age-related hearing loss

**DOI:** 10.3389/fnagi.2026.1796527

**Published:** 2026-06-02

**Authors:** Stefan Elmer, Martin Meyer, Nathalie Giroud

**Affiliations:** 1 Department of Computational Linguistics, University of Zurich, Zurich, Switzerland; 2 Competence Center Language and Medicine, University of Zurich, Zurich, Switzerland; 3 Department of Child and Adolescent Psychiatry, Neurosciences of Communication, Language, and Cognition Research Group, University of Zurich, Zurich, Switzerland; 4 Department of Adult Psychiatry and Psychotherapy, University Hospital of Psychiatry Zurich, University of Zurich, Zurich, Switzerland; 5 Department of Comparative Language Science, University of Zurich, Zurich, Switzerland; 6 Neuroscience Center Zurich, ETH Zürich and the University of Zurich, Zurich, Switzerland; 7 Center for the Interdisciplinary Study of Language Evolution (ISLE), University of Zurich, Zurich, Switzerland; 8 Cognitive Psychology Unit, Alpen-Adria University Klagenfurt, Klagenfurt, Austria

**Keywords:** aging, EEG, fast Fourier transform, frequency-tagging, multi-talker babble noise, naturalistic speech, pure-tone hearing loss, syllabic rate

## Abstract

**Introduction:**

Healthy aging is often accompanied by a loss of hearing sensitivity and a reduced ability to understand speech in both quiet and noisy environments. From a physiological perspective, neural oscillations are thought to fundamentally contribute to parse the speech signal into meaningful units and to extract linguistically relevant information at multiple hierarchical levels. Given that syllables are essential structural elements of the speech signal that recur with quasi-rhythmic periodicity and are firmly anchored in slow amplitude modulations of the acoustic envelope, we exploited the potential of frequency-tagging to examine effects of pure-tone hearing loss and background noise on syllable representation at the neural level.

**Methods:**

We used electroencephalography and compared peak amplitudes in a narrow-band frequency range corresponding to the syllabic speech rate in two samples of older adults with normal hearing (*N=44*) and mild-to-moderate age-related hearing loss (*N=38*) while participants listened to naturalistic sentences presented in quiet or embedded in multi-talker babble noise.

**Results:**

The behavioral indices confirmed that the multi-talker babble noise condition was more demanding, and that hearing loss was generally associated with reduced intelligibility, but not with lower comprehension. Notably, the effects of background noise and hearing loss were also clearly represented at the cortical level, with similar neural response properties. In fact, both acoustically and physiologically impoverished speech signals were expressed in a weaker neural representation of syllables.

**Discussion:**

These results provide neurophysiological evidence for an analogous but qualitatively different neural attenuation of syllable representation under adverse listening conditions and in individuals with pure-tone hearing loss.

## 1 Introduction

Syllables are salient acoustic and linguistic features of the speech signal that recur with a quasi-rhythmic temporal regularity of approximately 4–8 Hz across different languages (Ding et al., 2017; Meyer, 2018; Poeppel and Assaneo, 2020). These linguistic units are also clearly reflected in the temporal envelope of speech, in which local maxima roughly correspond to the syllable production rate and minima to the respective boundaries (Coupé et al., 2019; Ding et al., 2017; Pellegrino et al., 2011; Poeppel and Assaneo, 2020; Varnet et al., 2017). Thus, it is not surprising that the center frequency of the speech envelope correlates fairly well with the syllable rate, as it only confirms that syllabic information is clearly represented in the speech signal (Abrams et al., 2009; Aiken and Picton, 2008; Zhang et al., 2023). From a linguistic perspective, there are several reasons to believe that the decoding of syllables from continuous speech significantly contributes to both speech intelligibility and comprehension (Ghitza, 2013; Giraud and Poeppel, 2012; Versfeld and Dreschler, 2002). Speech intelligibility refers to perceptual identification and is commonly assessed by the number of words or phonemes that a listener correctly recognizes (Baese-Berk et al., 2023), while speech comprehension implies access to higher-level linguistic representations which are needed to understand the conceptual meaning of spoken utterances (Fontan et al., 2015; Wilson and Spaulding, 2010). A first argument is rooted in the knowledge that suppression (Drullman et al., 1994) or flattening (Ghitza, 2012) of the speech envelope leads to poor comprehension, most likely because the impoverished syllable rhythm in the amplitude modulation spectrum disrupts syllabic parsing (Ghitza, 2012; Malaia and Wilbur, 2020). However, a listener is easily able to understand spectrally degraded speech as long as the envelope is preserved (Shannon et al., 1995). Interestingly, comprehension has also been shown to decrease when the syllabic rate deviates from natural speech, and more than about 12 syllables per second are presented (Ghitza, 2013; Giraud and Poeppel, 2012; Versfeld and Dreschler, 2002). Nevertheless, if additional periodic silent pauses are added to the time-compressed speech signal to restore the natural syllable rhythm with an envelope modulation of about 4 Hz, comprehension remarkably improves (Ghitza and Greenberg, 2009).

The neural representation of acoustic and linguistic information from spoken language has been proposed to be mediated by endogenous neural oscillations that adjust their high-excitability phase to the input signal (Meyer, 2018; Poeppel and Assaneo, 2020), by exogeneous phase-resetting mechanisms (Zoefel et al., 2018), or even by rhythmical stimulus-evoked activity (Elmer et al., 2021; Zoefel et al., 2018). Although there is still disagreement about the advantages, caveats and limitations of these neurophysiological principles, they are all potentially useful for capturing relevant features of the acoustic spectrum and for decomposing speech into discrete linguistic units such as phonemes, syllables, words, and sentences (Christiansen and Chater, 2016; Giraud and Poeppel, 2012; Kösem and van Wassenhove, 2017). Given that neural oscillations in the theta frequency range (∼ 4–7 Hz) overlap with syllable rhythm, this specific neural metric could represent an intuitively appealing physiological mechanism that facilitates the segmentation of the speech signal into single units (Giroud et al., 2023; Guilleminot and Reichenbach, 2022; Howard and Poeppel, 2012; Luo and Poeppel, 2007; Peelle et al., 2013). Such a syllabic parsing operation has been inferred, for example, based on cross-correlations between the speech envelope and neural oscillations (Doelling et al., 2014; Millman et al., 2015; Schmitt et al., 2022), inter-trial phase coherence measures (Batterink and Paller, 2017; Elmer et al., 2021), or on frequency-tagging procedures restricted to a specific frequency and directly related to the stimulus presentation rate (Buiatti et al., 2009; Ramos-Escobar et al., 2021). Direct evidence for the ability of theta oscillations to follow syllable rhythms in speech has been presented by several authors (Ding and Simon, 2014; Doelling et al., 2014; Etard and Reichenbach, 2019; Pefkou et al., 2017). For example, Pefkou et al. (2017) used EEG and exposed subjects to comprehensible and incomprehensible time-compressed speech and showed that neural phase in the theta band consistently reflected syllable rate, even when speech became too fast to be intelligible. Furthermore, theta power increased linearly with syllable rate, providing evidence for a relationship between theta oscillations and stimulus-driven syllable representation. Another argument for a link between theta activity and syllable representation comes from frequency-tagging studies that examined speech segmentation and word learning based on transition probabilities between adjacent syllables (Batterink and Paller, 2017; Buiatti et al., 2009; Elmer et al., 2021). In one of these studies, Buiatti et al. (2009) measured EEG while participants listened to sequences of either random syllables or trisyllabic pseudowords. The random streams elicited clear spectral power peaks in the theta frequency range corresponding to the syllabic rate, while the pseudoword condition was associated with the appearance of power maxima in the delta frequency corresponding to the word rate. Because syllables have clear acoustic boundaries (Ding et al., 2017) and exhibit a rhythmic temporal regularity of about 4–8 Hz in different languages (Ding et al., 2017; Meyer, 2018; Poeppel and Assaneo, 2020), they are thought to elicit strong auditory responses in the theta range that can be measured at the neural level.

Pure-tone hearing loss (HL) or presbycusis is one of the most common and widespread chronic conditions, which is particularly pronounced in older individuals and characterized by poorer hearing in the high frequency range of the acoustic spectrum (Gates and Mills, 2005). According to large cohort studies, nearly 45%–60 % of individuals in the age-range of 60–90 years have an average pure-tone HL of 20 dB or more at 0.5–4 kHz in the better ear (Cruickshanks et al., 1998; Gates et al., 1990; Homans et al., 2017; Moscicki et al., 1985). However, HL is not only manifested in a shift of pure-tone hearing thresholds, but also leads to significant impairment of speech intelligibility and comprehension on several linguistic levels (Giroud et al., 2017; Hornsby and Ricketts, 2003; Neuschwander et al., 2023; Schmitt et al., 2022; Tremblay et al., 2021). Such speech processing difficulties in older adults with pure-tone HL have been shown to occur already at the syllable scale and are manifested, for example, in atypical event-related potentials (ERP) in response to naturalistic syllables or items manipulated in the temporal or spectral dimension (Elmer et al., 2023a; Oron et al., 2019; Strouse et al., 1998; Tremblay et al., 2002). In this context, what is commonly found are increased N100/P200 ERP amplitudes and longer latencies, possibly reflecting neuro-functional compensation mechanisms which are required to deal with reduced hearing acuity, especially in adverse listening conditions (Bartrés-Faz and Arenaza-Urquijo, 2011; Elmer et al., 2023a; Oron et al., 2019).

Given that syllables are firmly anchored in the temporal speech envelope (Ding et al., 2017; Zhang et al., 2023), neural speech tracking can be understood as a rough measure of syllable representation at the neural level. Currently, only a few studies have exploited the potential of this technique to provide indirect evidence for a distinctive encoding of syllabic information in individuals affected by pure-tone HL while listening to speech in quiet as well as in noisy environments (Decruy et al., 2020; Fuglsang et al., 2020; Petersen et al., 2017; Schmitt et al., 2022). Relying on cross-correlations between the electroencephalogram (EEG) and the speech envelope, most (Decruy et al., 2020; Fuglsang et al., 2020; Schmitt et al., 2022) but not all (Petersen et al., 2017) studies found an increased correspondence between the two time series in individuals affected by HL. These results have typically been interpreted as reflecting compensatory mechanisms whereby individuals with HL rely more heavily on slow acoustic modulations to improve speech processing (Schmitt et al., 2022). In light of this, it is worth noting that in most neural tracking studies the maximal correspondence between the EEG signal and the speech envelope occurred at time lags of approximately 100 and 200 ms. Such a time-specific delay is indeed not surprising, as it corresponds to the temporal evolution of the N100 and P200 ERP components in response to syllabic units (Bosnyak et al., 2004; Liégeois-Chauvel et al., 1994).

In the present study, we applied a simple EEG metric to examine periodic brain responses restricted to a specific frequency range and directly related to the syllable rate in natural speech (Batterink and Paller, 2017; Buiatti et al., 2009; Nozaradan, 2014). With this purpose in mind, we used a frequency-tagging approach (Buiatti et al., 2009) as a valuable technique to measure neural syllable representation while two matched samples of older individuals (age-range 63–80 years) with hearing in the normative range (HL < 20 dB) or affected by pure-tone HL (HL > 20 dB) listened to naturalistic sentences presented in quiet and multi-talker babble noise. Based on previous studies, we predicted that HL and background noise would be manifested in lower speech intelligibility and comprehension (Ching and Dillon, 2013; Huang et al., 2023; Peelle et al., 2011; Schmitt et al., 2022; Shafiro et al., 2015). Furthermore, we expected that these behavioral indices would be accompanied by different narrow-band peak amplitudes in the frequency range corresponding to the syllable rate (Batterink and Paller, 2017; Buiatti et al., 2009). Finally, we also tested possible associations between the degree of HL, behavioral indices and neural metrics using explorative correlation analyses.

## 2 Materials and methods

Some of the materials included in this work have already been used in previous publications by our group (Elmer et al., 2023a,b; Schmitt et al., 2022). In particular, the intelligibility and comprehension tasks as well as the auditory stimuli are the same as in Schmitt et al. (2022), whereas the psychometric tests and the audiometry were described in detail in a previous publication by Elmer et al. (2023b). In addition, the EEG data were collected with the same device as described in a previous work (Elmer et al., 2023a). Accordingly, the descriptions of the procedures used in this research were partially adapted from our previous publications.

### 2.1 Participants

In the present study, we re-evaluated the EEG data from a previous work by our group (*N* = 101), the aim of which was to assess the relationships between pure-tone HL and neural speech tracking (Schmitt et al., 2022). However, due to noisy EEG recordings, the data from 6 participants in the control group (CG, without HL and without tinnitus) and from 11 subjects in the HL group (non-tinnitus and hearing loss, NTHL) had to be excluded from the analyses. In addition, 1 participant from the CG and 1 from the NTHL group had to be dismissed due to a technical problem with the response box. The remaining sample of participants (*N* = 82) was subdivided into two cohorts of older individuals (CG and NTHL) according to a threshold of 20 dB HL (Humes, 2020). This procedure resulted in a group with hearing in the normative range (CG, *n* = 44, HL < 20 dB, age range = 64–80 years, *M* = 69.59, SD = 3.56, 22 female) and one with mild-to-moderate pure-tone HL (NTHL, *n* = 38, HL > 20 dB, age range = 63–79 years, *M* = 71.02, SD = 4.11, 19 female). All participants were consistently right-handed (Annett, 1970), native German speakers, and did not report past or present neurological, psychological or psychiatric impairments. Furthermore, none of the participants were exposed to a second language before the age of 7 years or played a musical instrument for more than 10 h per week. All participants gave informed written consent in accordance with the procedures of the local ethics committee (“Kantonale Ethikkommission Zürich”) and the declaration of Helsinki and were paid for participation.

### 2.2. Cognitive capabilities

To rule out that the two groups differed in general cognitive performance, we conducted a series of psychometric tests and examined fluid and crystallized intelligence, processing speed and working memory. Fluid intelligence was screened using the KAI test (“Kurztest für Allgemeine Basisgrössen der Informationsverarbeitung”) (Lehrl et al., 1992). This procedure consisted of reading aloud meaningless sequences of 20 letters as quickly as possible, and of repeating auditory-presented letters and digits of increasing length (up to nine items). Otherwise, crystallized intelligence was assessed using the MWT-B test (“Mehrfachwahl-Wortschatz-Intelligenztest,” version B) (Lehrl, 1977) which included 37 trials ordered as a function of difficulty level, and the participants had to select the unique word with a meaning from a set of five words that included four pseudowords. Both the KAI and MWT-B tests enable to estimate general intelligence in a short time, and have previously been shown to correlate fairly well (*r* ∼ 0.7) with the global intelligence quotient in healthy adults (Lehrl et al., 1992). Furthermore, processing speed was screened by means of the ZST (“Zahlen-Symbol-Test”) test which is included in the Wechsler adult intelligence scale (WAIS) (Wechsler, 1981). This digit-symbol-coding test is a speed-dependent procedure in which simple graphical symbols have to be associated with predetermined numbers in the range of 1–9 as quickly as possible according to an assignment key printed on the test sheet. The test value is calculated based on the number of symbols that are correctly paired with the corresponding numbers in the time range of 120 s. Finally, working memory (WM) was evaluated using a numerical n-back task implemented in the TAP (“Testbatterie zur Aufmerksamkeitsprüfung”) test battery (Zimmermann and Fimm, 2002). In this task, participants were visually confronted with numbers displayed on a screen and had to decide whether each individual item matched the previous one or the next-to-last. All the psychometric tests were compared between the two groups according to normative T-values.

### 2.3 Pure-tone audiometry

**FIGURE 1:**
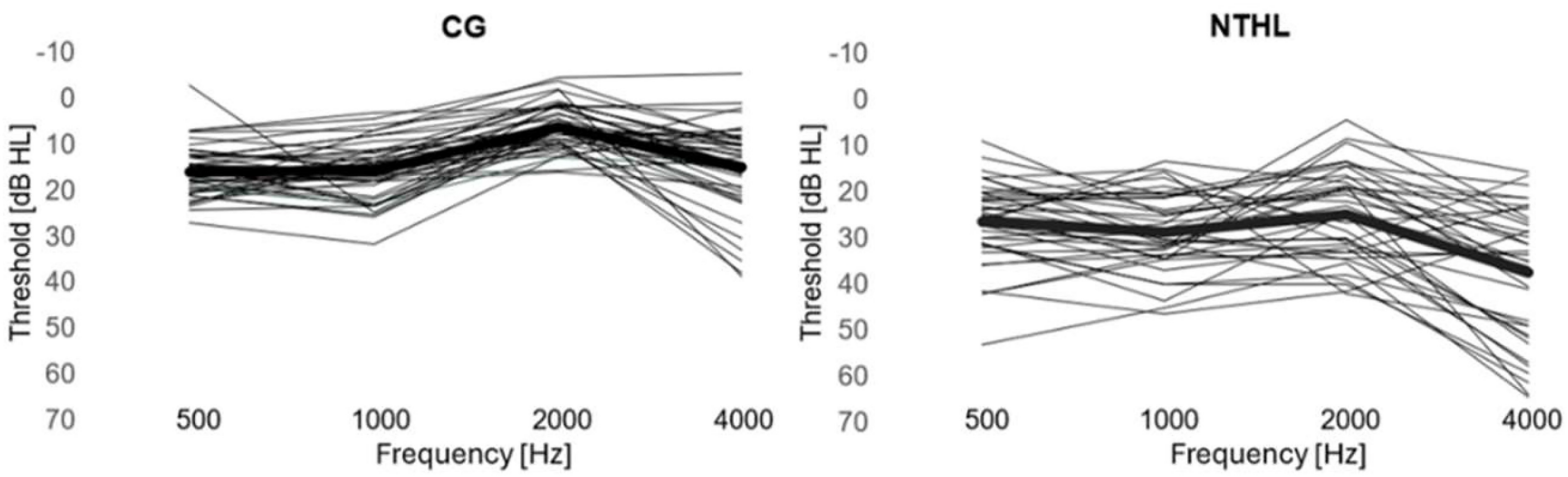
Audiometric profiles. Pure-tone hearing thresholds (dB HL) in the range of 0.5–4 kHz are depicted separately for the control group (CG, left side) and the non-tinnitus group with hearing loss (NTHL, right side). Each thin line represents an individual participant, whereas the thick line depicts the mean of the sample. dB, decibel; HL, hearing loss.

Audiometry was conducted separately for both ears to determine the degree of peripheral HL and consisted of detecting pure tones presented for 250 ms at 500, 1,000, 2,000, and 4,000 Hz. Thereby we used the same in-house MATLAB-based procedure as described in previous studies of our group (Elmer et al., 2023a; Giroud et al., 2018; Schmitt et al., 2022). Furthermore, to provide a global assessment of hearing acuity for each participant, we used pure-tone averages (PTA) by computing mean hearing thresholds across the two ears and the octave frequencies in the range of 500–4,000 Hz. According to this procedure, all participants demonstrated nearly symmetrical pure-tone thresholds for both ears (<15 dB interaural threshold difference). Pure-tone HL loss and thus group assignment (CG and NTHL) was defined in accordance with the WHO criteria (Humes, 2020) as follows: normal hearing ≤ 20; slight/mild HL = 20–35; moderate HL = 35–50; moderately severe HL = 50–65; severe HL = 65–80; profound HL ≥ 80 dB HL. The CG consisted of older individuals with hearing acuity in the normative range (HL < 20 dB, PTA = 13.22, SD = 4.21), whereas the NTHL exhibited mild-to-moderate HL (HL > 20 dB, PTA = 29.39, SD = 7.37). The audiometric profiles of both groups are shown in Figure 1.

### 2.4 Auditory stimuli and tasks

During the EEG experiment, the participants heard sentences spoken in standard German by a trained female speaker. To ensure neutrality in terms of content, all sentences were based on EU regulations.^1^ Furthermore, the sentences were either presented in quiet or in a multi-talker babble environment, and the serial order of the conditions was counterbalanced. Multi-talker babble noise consisted of eight randomly selected and superimposed sentences from the same speaker, and silent pauses were edited out to make “hearing in the gap” impossible. Specifically, the same speaker was used for both the target speech and the background babble sentences. We chose this approach deliberately in order to maintain strict experimental control over acoustic and speaker-related features across conditions. For each of the two conditions 30 sentences with an average length of 10.34 s (range = 8.41–12.35) were presented at 70 dB SPL so that they could be easily heard by all participants. The signal-to-noise ratio (SNR) of the multi-talker babble condition was set to 0, meaning that the intensities of the signal and noise were equal. This SNR was previously tested in a pilot study to ensure high performance in speech comprehension. The noise faded in 1.5 s after the start of the sentence to make it easier to focus on the target signal and lasted until the end of the sentence. Importantly, after each sentence, participants also completed an intelligibility task consisting of deciding whether a 300 ms speech excerpt was part of the preceding sentence or not by clicking the corresponding mouse button (yes = left, no = right), a procedure adapted from previous studies conducted by our group (Frei et al., 2024; Schmitt et al., 2022). The short excerpts presented during the intelligibility task were randomly selected from the last 3 to 0.3 s of each sentence to guarantee minimal working memory load. Otherwise, speech comprehension was tested by presenting a four-alternative forced-choice question on the screen after every fifth sentence, and the participants had to select the correct answer by pressing the correct number on the keyboard (in the range of 1–4). The comprehension questions referred only to the most recently presented sentence and not to the entire set of preceding sentences. The experiment began with a training session in which participants performed the intelligibility and comprehension tasks in silence. After each training trial, visual feedback was provided on a screen, and the training trials were administered until the participant performed without errors to ensure that the task was correctly understood. To examine periodic brain responses restricted to syllable frequency, the syllabic rate of the sentences was automatically estimated in Praat (version 6.1.40) (Boersma and Weenick, 2020), using de Jong and Wempe’s (2009) algorithm for syllable nuclei detection. Based on this analysis, syllable frequency was in the range of 4–5.6 Hz, with a mean of 4.66 syllables per second across all sentences.

### 2.5 EEG data acquisition and processing

**FIGURE 2:**
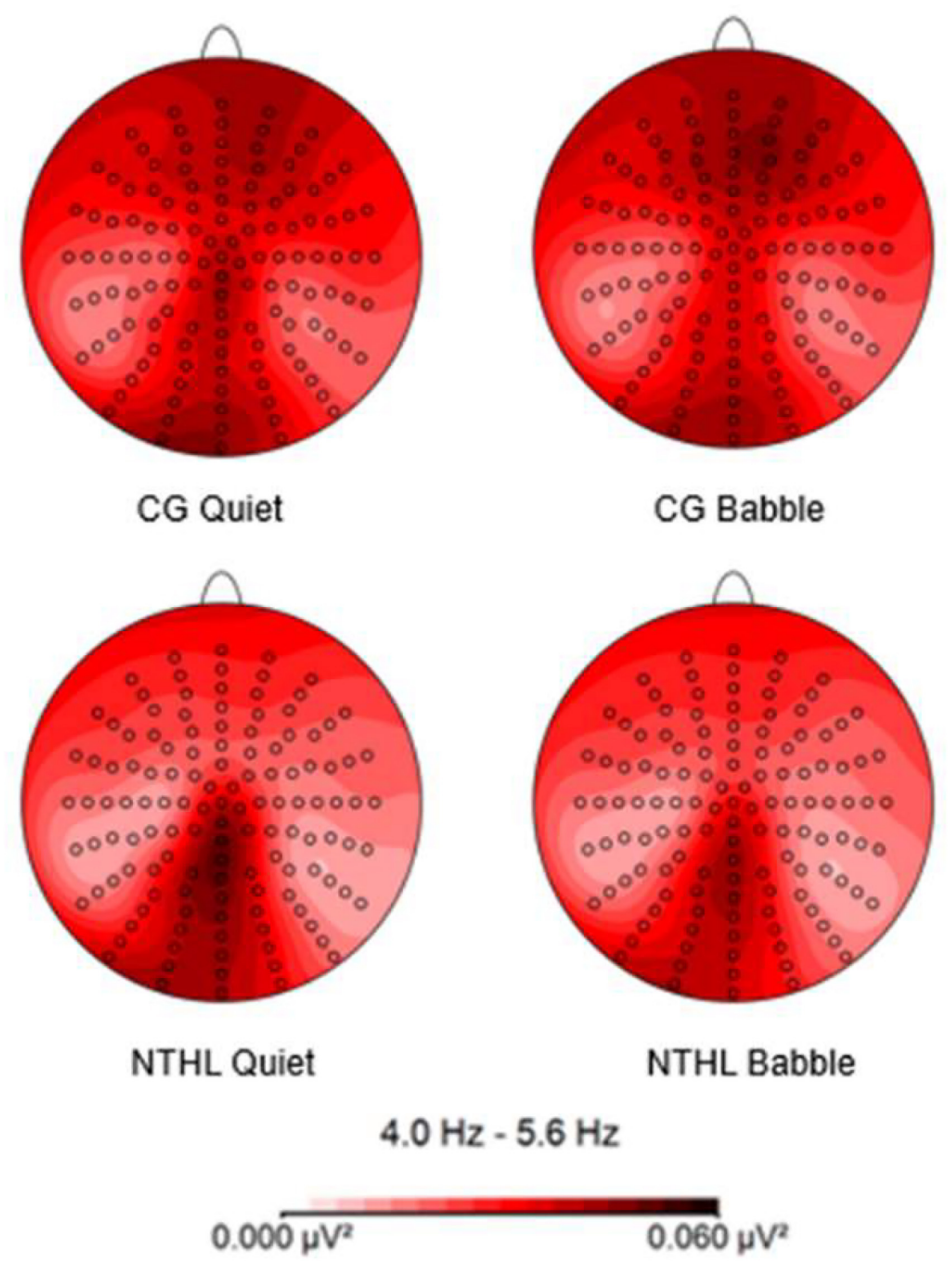
Topographic power distribution maps corresponding to the mean power in the range of the syllable rate (4–5.6 Hz) are depicted separately for the two groups (upper row, CG; lower row, NTHL) and the two conditions (quiet, left; babble, right).

EEG was recorded at a sampling rate of 512 Hz using a BIOSEMI 128-channel system (ActiveTwo, BioSemi B.V., Amsterdam, Netherlands) and filtered online with a bandpass filter of 0.1–100 Hz. Eye movements were monitored with two electrodes placed below the eyes and electrode impedances were kept below 20 kΩ. All pre-processing steps were performed with the Brain Vision Analyzer software package (Version 2.0.4, BrainProducts, Munich, Germany). In particular, the data were re-referenced offline to the linked mastoids, bandpass filtered in the range of 0.5–50 Hz using a zero-phase shift Butterworth filter (order 8) and a band-stop Notch filter at 50 Hz, and noisy channels were interpolated. Eye blinks and saccades were corrected using an Independent Component Analysis (ICA) (Jung et al., 2000), whereas remaining muscle artifacts were removed from -200 ms before to 200 ms after events using an automatic raw data inspection if a voltage gradient criterion of 50 μV/ms or an amplitude criterion of ±100 μV was exceeded. For the frequency-tagging analyses, the EEG data were additionally filtered in the range of 4–5.6 Hz by mean of a zero-phase shift Butterworth filter (order 8). This procedure was used to limit the frequency range of interest to brain responses directly related to syllable presentation rate. Afterwards, brain responses to the sentences of both conditions were segmented from −100 to 8,000 ms (shortest sentence duration) and baseline corrected from −100 to 0 ms. Individual sentences were then additionally segmented from 3,000 to 8,000 ms to minimize onset responses elicited by the fade in of the noise 1.5 s after the beginning of the sentences and subjected to fast Fourier transforms (FFT) to further decompose the frequency spectrum within the syllable range with a resolution of 0.125 Hz. In a next step, the FFT-transformed data of the individual sentences were averaged separately for each of the two conditions, and maximal power spectra values were extracted at three anterior (F3, Fz, F4), three central (C3, Cz, C4), and three posterior (P3, Pz, P4) electrodes. This reduced pool of nine electrodes was used to cover brain activity along both the anterior-posterior and lateral topographical axes, and was selected based on the topographic distribution maps (Figure 2), on a previous publication of our group focusing on neural envelope tracking with the same sentences (Schmitt et al., 2022), as well as on previous literature indicating a shift of brain dynamics along the two axes as a function of aging (Pfefferbaum et al., 1980; Sandman and Patterson, 2000) and HL (Campbell and Sharma, 2013; Giroud et al., 2017). In addition, we wanted to avoid the problem of multiple comparisons, because as the number of comparisons increases, the correction becomes more conservative, potentially leading to a loss of statistical power and an increased risk of type II errors (false negatives).

### 2.6 Statistical analyses

All data were analyzed using the IBM SPSS Statistics 22 software (SPSS, an IBM company, Armonk, New York, United States). The biographical, audiometric and psychometric data were compared between the two groups using *t*-tests for independent samples (Bonferroni-corrected for multiple comparisons). Otherwise, all comparisons performed with the EEG and behavioral data were conducted using analyses of variance (ANOVAs, repeated measures) with specific factors of interest for each model. Significant main and interaction effects were further inspected by means of ANOVAs or *post-hoc t*-tests (two-tailed). The behavioral data of the intelligibility and comprehension tasks were evaluated by means of 2 × 2 ANOVAs with the factors group (CG and NTHL) and conditions (quiet and multi-talker babble noise). The data of the intelligibility task were transformed into d-prime values (Stanislaw and Todorov, 1999), whereas for the comprehension task we analyzed the percentage of correct responses. The EEG data were examined by means of a four-way 2 × 3 × 3 × 2 ANOVA with the factors condition, anterior-posterior, laterality and group. These statistical procedures are widely used due to their robustness to moderate deviations from normality, particularly in sufficiently large and balanced samples. Under such conditions, the sampling distribution of the mean approximates normality, supporting the validity of both the *t*-test and the F-test (Field, 2018; Schmider et al., 2010). Simulation studies further demonstrate that both tests maintain stable Type I error rates under non-normal and skewed distributions in typical research settings (Blanca et al., 2017; Schmider et al., 2010). Based on the results, additional exploratory correlation analyses were conducted within the full sample of participants and separately for the two groups using Spearman’s rho (two-tailed). All *post-hoc* tests and correlation analyses were corrected for multiple comparisons using the Bonferroni procedure.

## 3 Results

### 3.1 Age, audiometry and cognitive abilities

As a result of dividing participants into two groups based on hearing acuity, the evaluation of the PTAs confirmed that the CG had better hearing than the NTHL group [*t*_(80)_ = −12.397, *p* < 0.001; M_*CG*_ = 13.22, SD_*CG*_ = 4.21, M_*NTHL*_ = 29.39, SD_*NTHL*_ = 7.38; Figure 1]. Furthermore, the two groups did not differ in age [*t*_(80)_ = −1.694, *p* = 0.094; M_*CG*_ = 69.59, SD_*CG*_ = 3.56, M_*NTHL*_ = 71.02, SD_*NTHL*_ = 4.11] or in general cognitive abilities, as measured by the KAI [*t*_(80)_ = 0.194, *p* = 0.847; M_*CG*_ = 52.80, SD_*CG*_ = 8.10, M_*NTHL*_ = 52.45, SD_*NTHL*_ = 8.11], MWT-B [*t*_(80)_ = −0.495, *p* = 0.622; M_*CG*_ = 66.75, SD_*CG*_ = 8.41, M_*NTHL*_ = 67.67, SD_*NTHL*_ = 8.41], ZST [*t*_(80)_ = −0.873, *p* = 0.385; M_*CG*_ = 47.50, SD_*CG*_ = 5.80, M_*NTHL*_ = 48.59, SD_*NTHL*_ = 5.51], and WM tests [*t*_(80)_ = 0.765, *p* = 0.446; M_*CG*_ = 49.36, SD_*CG*_ = 8.65, M_*NTHL*_ = 47.89, SD_*NTHL*_ = 8.68].

### 3.2 Behavioral data

**FIGURE 3:**
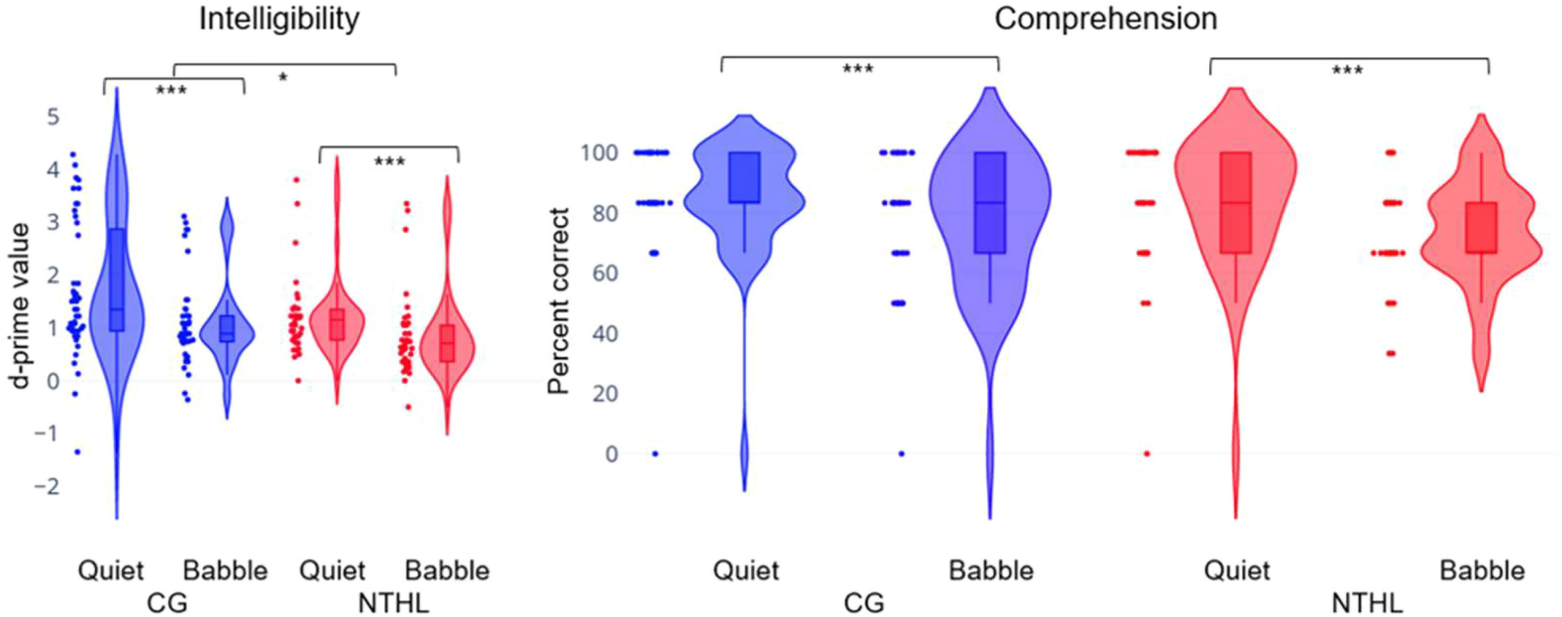
Density distributions of the behavioral data of the intelligibility (left side, d-prime values) and comprehension (right side, percentage of correct responses) tasks with single-subject values. The boxes indicate the interquartile range with upper and lower fence, whereas the line within the box depicts the mean. CG, control group; NTHL, non-tinnitus hearing loss group. *p < 0.05, ***p < 0.001.

In an initial analysis, d-prime values of the intelligibility task and the percentage of correct responses in the comprehension task were tested against chance level (d-prime = 0, % of correct responses = 25) using separate one-sample *t*-tests for the two groups, the two conditions and the two tasks (Bonferroni-corrected *p*-value for two tests = 0.025). According to this procedure, all behavioral metrics were above chance level (CG and NTHL, all *p*-values < 0.001, Figure 3). The evaluation of the d-prime values of the intelligibility task by means of a 2 × 2 ANOVA (two groups and two conditions) yielded main effects of condition [*F*_(1, 80)_ = 11.899, *p* < 0.001, partial eta^2^ = 0.129, Figure 3, left side] and group [*F*_(1, 80)_ = 5.844, *p* = 0.018, partial eta^2^ = 0.068, Figure 3, left side], while the interaction between the two variables did not reach significance [*F*_(1, 80)_ = 0.789, *p* = 0.377, partial eta^2^ = 0.010]. The main effect of condition originated from higher d-prime values in the quiet compared to the multi-talker babble condition, whereas the main effect of group was driven by an overall better performance in the CG compared to the NTHL group. Otherwise, the statistical analysis of the speech comprehension task by a 2 × 2 ANOVA (two groups and two conditions) revealed a main effect of condition [*F*_(1, 80)_ = 12.616, *p* < 0.001, partial eta^2^ = 0.136, Figure 3, right side], whereas the main effect of group [*F*_(1, 80)_ = 0.320, *p* = 0.573, partial eta^2^ = 0.004] as well as the interaction between the two variables [*F*_(1, 80)_ = 0.117, *p* = 0.733, partial eta^2^ = 0.001] did not reach significance. The main effect of condition was related to better comprehension in the quiet compared to the multi-talker babble condition.

### 3.3 Electrophysiological data

The evaluation of narrow-band peak amplitudes in the frequency range corresponding to the syllable rate (4–5.6 Hz) by means of a 2 (conditions) × 3 (anterior-posterior) × 3 (laterality) × 2 (groups) ANOVA revealed significant main effects of anterior-posterior (F_(2, 160)_ = 7.880, *p* < 0.001, partial eta^2^ = 0.090) and laterality [F_(2, 160)_ = 56.904, *p* < 0.001, partial eta^2^ = 0.416], as well as anterior-posterior × group [*F*_(1.707, 136.596)_ = 6.169, *p* = 0.004, partial eta^2^ = 0.072], laterality × group [*F*_(1.227, 98.166)_ = 4.688, *p* = 0.026, partial eta^2^ = 0.055], condition × anterior-posterior [*F*_(1.761, 140.914)_ = 6.616, *p* = 0.003, partial eta^2^ = 0.076], condition × laterality [*F*_(1.128, 90.256)_ = 6.356, *p* = 0.011, partial eta^2^ = 0.074], anterior-posterior × laterality [*F*_(4, 320)_ = 37.763, *p* < 0.001, partial eta^2^ = 0.321], and condition × anterior-posterior × laterality [*F*_(1.606, 128.509)_ = 6.691, *p* = 0.003, partial eta^2^ = 0.077] interaction effects (Tables 1, 2).

**TABLE 1:** Overview of significant main and interaction effects obtained from a 2 (conditions) × 3 (anterior-posterior) × 3 (laterality) × 2 (groups) ANOVA on narrow-band peak amplitudes in the frequency range corresponding to the syllable rate.

Main and interaction effects	Test statistic	*P*-value
Anterior-posterior	*F*_(2, 160)_ = 7.880	<0.001
Laterality	*F*_(2, 160)_ = 56.904	<0.001
Anterior-posterior × group	*F*_(1.707, 136.596)_ = 6.169	0.0040
Laterality × group	*F*_(1.227, 98.166)_ = 4.688	0.0260
Condition × anterior-posterior	*F*_(1.761, 140.914)_ = 6.616	0.0030
Condition × laterality	*F*_(1.128, 90.256)_ = 6.356	0.0110
Anterior-posterior × laterality	*F*_(4, 320)_ = 37.763	<0.001
Condition × anterior-posterior × laterality	*F*_(1.606, 128.509)_ = 6.691	0.0030

**TABLE 2:** Mean power spectra in the syllable-rate frequency range (4–5.6 Hz) and standard deviation (SD), reported separately for the two groups (CG and NTHL), the two conditions (quiet and multi-talker babble noise), and the electrode-based ROIs (anterior, central, posterior, left, middle, and right).

ROI/electrodes	Group and condition	Mean	SD
Anterior	CG quiet	0.0705	0.0613
	CG babble	0.0756	0.0646
	NTHL quiet	0.0274	0.0267
	NTHL babble	0.0292	0.0314
Central	CG quiet	0.0661	0.0340
	CG babble	0.0629	0.0309
	NTHL quiet	0.0725	0.0836
	NTHL babble	0.0538	0.0439
Posterior	CG quiet	0.0444	0.0240
	CG babble	0.0468	0.0272
	NTHL quiet	0.0491	0.0498
	NTHL babble	0.0408	0.0287
Left	CG quiet	0.0508	0.0348
	CG babble	0.0547	0.0351
	NTHL quiet	0.0339	0.0256
	NTHL babble	0.0298	0.0202
Middle Right	CG quiet	0.0787	0.0379
	CG babble	0.0778	0.0420
	NTHL quiet	0.0924	0.1016
	NTHL babble	0.0714	0.0566
	CG quiet	0.0515	0.0409
	CG babble	0.0529	0.0371
	NTHL quiet	0.0227	0.0198
	NTHL babble	0.0225	0.0199

**FIGURE 4:**
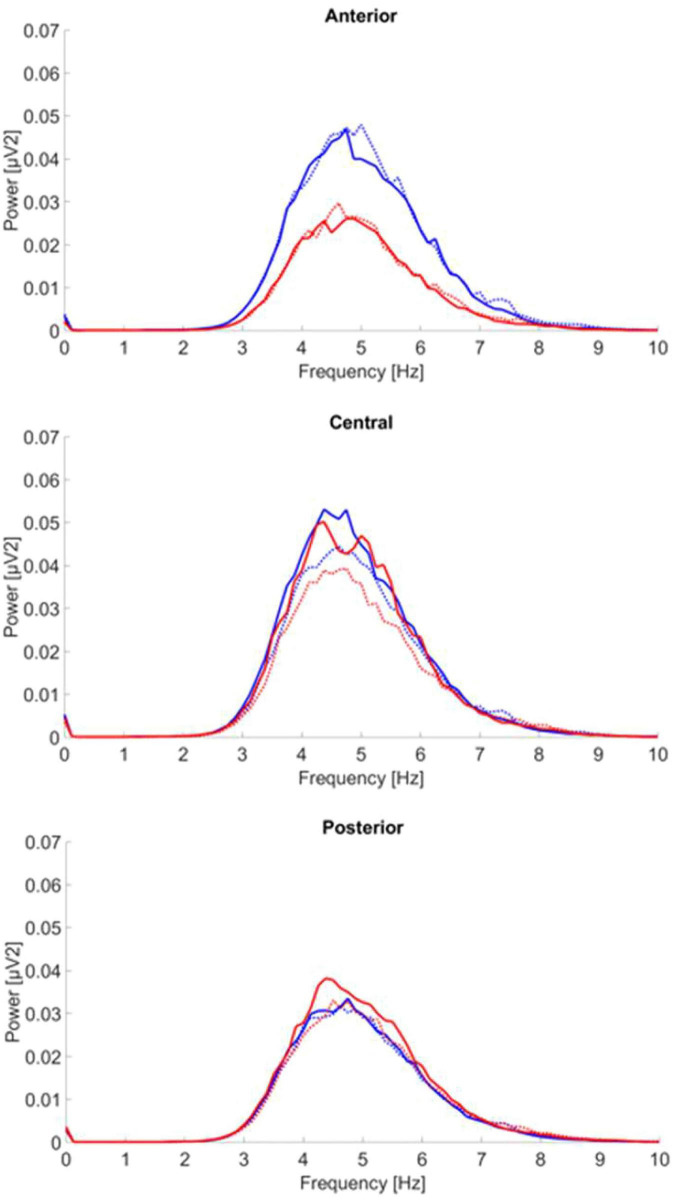
Power spectra in the range of the syllable rate (4–5.6 Hz) are depicted separately for the two groups (blue, CG; red, NTHL) and the two conditions (quiet, solid line; multi-talker babble noise, dashed line) at anterior (first row), central (second row), and posterior (third row) electrodes.

**FIGURE 5:**
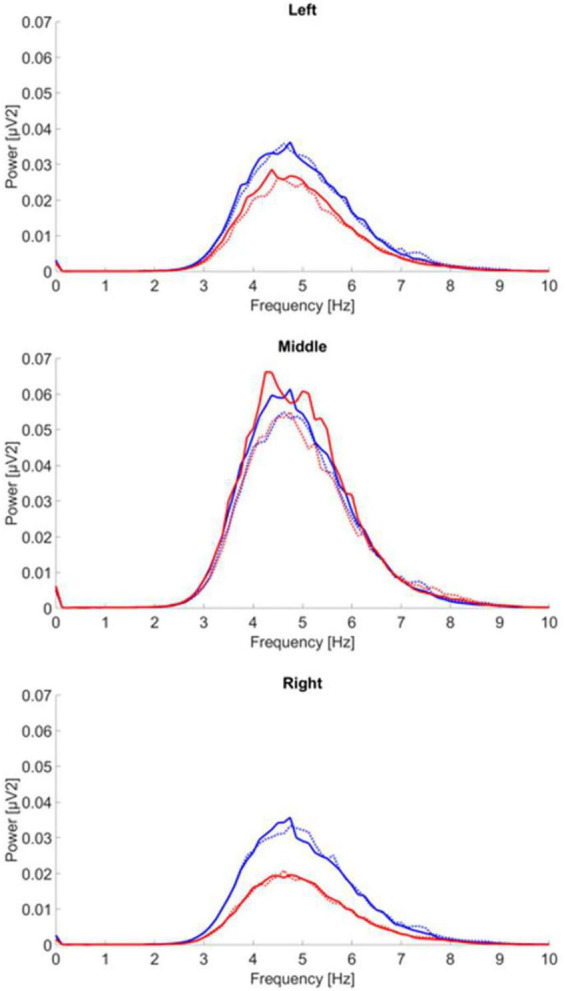
Power spectra in the range of the syllable rate (4–5.6 Hz) are depicted separately for the two groups (blue, CG; red, NTHL) and the two conditions (quiet, solid line; multi-talker babble noise, dashed line) at left (first row), middle (second row) and right (third row) electrode clusters.

The two main effects of anterior-posterior and laterality were decomposed using *t*-tests for dependent samples (Bonferroni-corrected *p*-value for three tests = 0.016). Based on this procedure, the anterior-posterior effect (Figure 4) was driven by increased power at central compared to anterior [*t*_(81)_ = −2.543, *p* = 0.013] and posterior electrodes [*t*_(81)_ = 4.829, *p* < 0.001; anterior vs. posterior *p* = 0.494], whereas the laterality effect (Figure 5) was related to increased amplitudes along the midline electrodes compared to left [*t*_(81)_ = −7.531, *p* < 0.001] and right [*t*_(81)_ = 7.483, *p* < 0.001; left vs. right *p* = 0.022]. Importantly, the two main effects of anterior-posterior and laterality were also modulated by group affiliation in the form of anterior-posterior × group and laterality × group interaction effects. These interactions were further inspected using *t*-tests for independent sample (Bonferroni-corrected *p*-value for three tests = 0.016). This approach revealed that the CG was characterized by increased power values at anterior [*t*_(80)_ = 2.475, *p* = 0.015; central *p* = 0.958; posterior *p* = 0.410, Figure 4] and right-sided electrodes [*t*_(80)_ = 2.707, *p* = 0.008; left *p* = 0.091; midline *p* = 0.545, Figure 5] compared to the NTHL group.

The condition × anterior-posterior and condition × laterality interactions were further inspected using *t*-tests for dependent sample (Bonferroni-corrected *p*-value for three tests = 0.016). The condition × anterior-posterior effect was driven by increased power values at central electrodes in the quiet compared to the multi-talker babble noise condition [*t*_(81)_ = 2.652, *p* = 0.010; anterior *p* = 0.252; posterior *p* = 0.213, Figure 4], whereas the condition × laterality interaction was related to increased power in the quiet compared to the multi-talker babble condition at midline electrodes [*t*_(81)_ = 2.259, *p* = 0.027; left *p* = 0.834; right *p* = 0.718, Figure 5], although the *post hoc* comparison did not survive the Bonferroni-correction.

**FIGURE 6:**
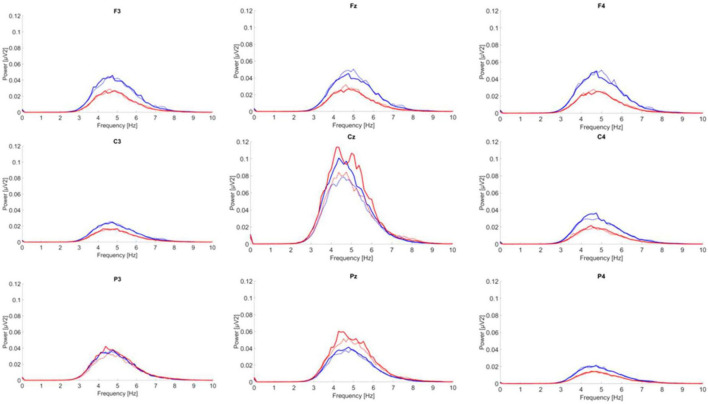
Power spectra in the range of the syllable rate (4–5.6 Hz) are depicted separately for the two groups (blue, CG; red = NTHL) and the two conditions (quiet, solid line; multi-talker babble noise, dashed line) at three anterior (F3, Fz, F4, first row), three central (C3, Cz, C4, second row), and three posterior (P3, Pz, P4, third row) electrodes.

The anterior-posterior × laterality and condition × anterior-posterior × laterality interactions were decomposed by means of ANOVAs (Bonferroni-corrected *p*-value for three tests = 0.016). Separate univariate ANOVAs run at anterior, central and posterior electrodes to disentangle the anterior-posterior × laterality interaction reached significance at central [F_(2, 162)_ = 47.662, *p* < 0.001, partial eta^2^ = 0.370) and posterior [F_(2, 162)_ = 31.744, *p* < 0.001, partial eta^2^ = 0.282) but not at anterior (*p* = 0.836) scalp sites. Additional *t*-tests for dependent samples (Bonferroni-corrected *p*-value for three tests = 0.016) showed increased power at electrode Cz compared to C3 [t_(81)_ = −7.330, *p* < 0.001] and C4 [t_(81)_ = 6.673, *p* < 0.001] as well as at C4 compared to C3 [t_(81)_ = −2.488, *p* = 0.015]. Furthermore, the same *post hoc* comparisons also showed increased power at electrode Pz compared to P3 [t_(81)_ = −2.844, *p* = 0.006] and P4 [t_(81)_ = 6.800, *p* < 0.001] as well as at electrode P3 compared to P4 [t_(81)_ = 6.191, *p* < 0.001]. All significant results are visible in Figure 6.

Finally, separate ANOVAs at anterior, central and posterior electrodes computed to disentangle the condition × anterior-posterior × laterality interaction (Bonferroni-corrected *p*-value for three tests = 0.016) yielded a significant condition × laterality effect at central electrodes only [*F*_(1.052, 85.186)_ = 7.147, *p* = 0.008, partial eta^2^ = 0.081; anterior *p* = 0.058; posterior *p* = 0.158]. Additional *t*-tests for dependent sample between the two conditions at central electrodes (Bonferroni-corrected *p*-value for three tests = 0.016) reached significance at sensor Cz only, with increased power spectra in the quiet compared to the multi-talker babble noise condition [*t*_(81)_ = 2.714, *p* = 0.008; C3 *p* = 0.267; C4 *p* = 0.224, Figure 6].

### 3.4 Correlation analyses

Since all correlations included PTA and behavioral measures of intelligibility and comprehension, only these variables were screened for normality to avoid an unnecessary increase in the number of statistical tests and the associated reduction in sensitivity due to multiple-comparison correction. According to the Kolmogorov-Smirnov test, PTA values were normally distributed, *D*(82) = 0.080, *p* = 0.200, whereas the behavioral measures of intelligibility and comprehension deviated significantly from normality (all *p* < 0.001). Given that PTA was also correlated with measures of intelligibility and comprehension, all correlation analyses were consistently performed using Spearman’s rho.

**FIGURE 7:**
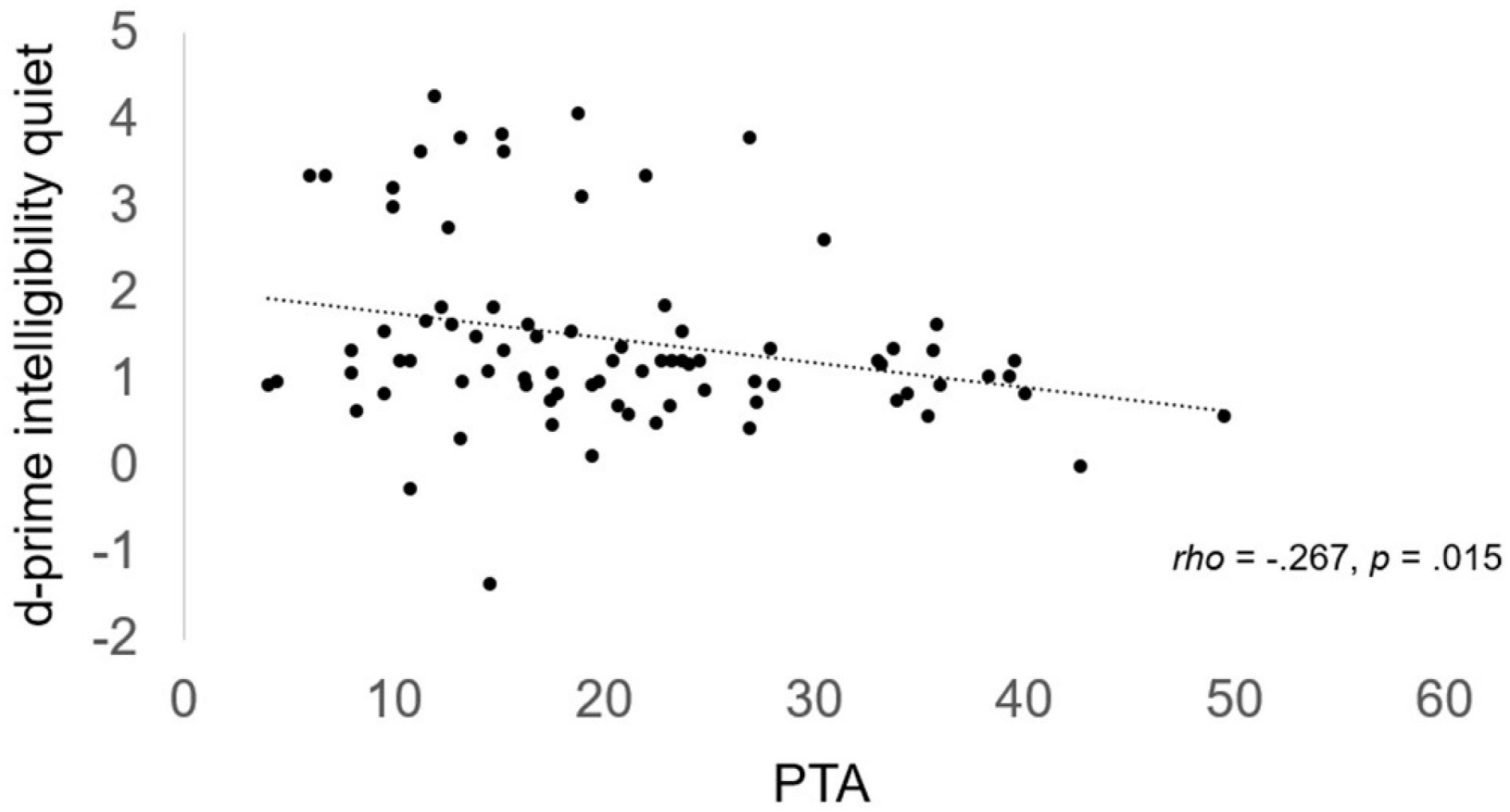
Significant negative correlation within the whole sample of participants between pure-tone averages (PTAs) and d-prime values of the intelligibility task in the quiet condition.

Based on previous literature (Ching and Dillon, 2013; Huang et al., 2023; Peelle et al., 2011; Schmitt et al., 2022; Shafiro et al., 2015), in a first step we examined associations between pure-tone HL (PTAs) and behavioral indices of intelligibility (d-prime) and comprehension (% of correct responses) in the quiet and multi-talker babble noise conditions within the whole sample of participants. With this purpose in mind, we calculated two correlations for intelligibility and two for comprehension (Bonferroni-corrected *p*-value for two tests = 0.025). This approach yielded a negative relationship between PTA and d-prime indices of the quiet condition, with higher PTAs associated with lower d-prime scores (rho = −0.267, *p* = 0.015, all other *p*-values > 0.059, Figure 7). Furthermore, since the behavioral data and the EEG metrics consistently showed effects of condition, within the whole sample of participants we also correlated intelligibility and comprehension scores with central, midline and Cz electrodes (Bonferroni-corrected *p*-value for three tests = 0.016). However, none of the correlations reached significance (all *p*-values > 0.126). Finally, due to the anterior-posterior × group and laterality × group interaction effects originating from increased power values in the CG compared to the NTHL group at anterior and right-sided electrodes, we performed additional separate correlations for the two groups. Specifically, we correlated mean EEG power across the quiet and multi-talker babble noise conditions with mean d-prime scores and percentage of correct responses of the two conditions (Bonferroni-correct *p*-value for two correlations = 0.025). None of the correlations reached significance, neither for the CG (all *p*-values > 0.382) nor for the NTHL group (all *p*-values > 0.311).

## 4 Discussion

### 4.1 General discussion

In the present study, we specifically focused on the neural strength of syllable representation as a function of background noise and hearing acuity while participants with and without HL listened to naturalistic sentences. To this end, we combined EEG with a frequency-tagging approach and transformed the quasi-periodic syllable rhythm into the spectral domain to extract representation-specific neural activity (Buiatti et al., 2009; Ramos-Escobar et al., 2021). In addition, we collected behavioral metrics of speech intelligibility and comprehension to determine which of the two factors is more likely to be influenced by HL and multi-talker babble noise. Results generally showed decreased comprehension and intelligibility when speech was presented in background noise. Furthermore, intelligibility, but not comprehension, was reduced overall in the NTHL group compared to the CG sample. Notably, the frequency-tagging approach consistently revealed neural attenuation of syllable representation in association with both acoustic and physiological hearing deterioration, with smaller EEG amplitudes in the multi-talker babble condition and in the NTHL cohort. Nevertheless, we also observed a topographic segregation of these effects, which suggests differences in the distribution of underlying neural activity and may reflect differential engagement of cognitive processes in the representation of syllables depending on the integrity of the speech signal and hearing status (Gordon-Salant, 1985; Shafiro et al., 2015).

### 4.2 Biographical, psychometric, and audiometric data

To compare neural syllable representation and behavioral data between participants with and without HL, we divided the elderly participants of a previous study by our group (Schmitt et al., 2022) into two cohorts according to WHO guidelines (Humes, 2020) and a HL threshold of 20 dB. Based on this group separation, all participants had approximately symmetrical pure-tone thresholds for both ears (<15 dB interaural threshold difference), but the CG consisted of older individuals with hearing acuity in the normal range (PTA = 13.22), while the NTHL group was characterized by mild-to-moderate HL with a PTA of 29.39 dB (Figure 1). As an addendum to the EEG and behavioral measures, we also tested fluid and crystallized intelligence to rule out between-group differences in general cognitive abilities. Furthermore, we assessed a small set of cognitive functions, namely processing speed and working memory, which often deteriorate with age (Albouy et al., 2017; Giroud et al., 2021) and HL (Heinrich et al., 2015; Quist-Hanssen et al., 1979). However, since the two groups did not differ in age or in the cognitive domains examined, it is unlikely that these variables influenced the results.

### 4.3 Behavioral data

We collected behavioral data on speech intelligibility and comprehension using a match-to-sample task (intelligibility) consisting of presenting short speech excerpts that were or were not part of the sentences, and a four-alternative forced-choice task (comprehension). Behavioral results showed that multi-talker background noise impaired both intelligibility and comprehension, which is not at all surprising and consistent with previous literature on a similar topic (Decruy et al., 2020; Kurthen et al., 2021; Schmitt et al., 2022). On the other hand, the behavioral measures also revealed a main effect of group that was limited to the intelligibility tasks and manifested in reduced performance in the NTHL group. Given that this main effect was not accompanied by a group × condition interaction, our results indicate that mild-to-moderate pure-tone HL generally affected speech intelligibility independently of background noise, at least under experimental conditions where the intensity of the signal and the noise was equal. However, this result should not be overinterpreted as previous studies have clearly demonstrated that intelligibility typically decreases with higher background noise levels, and we did not test additional SNRs (Pollack, 1958; Rajan and Cainer, 2008). Nevertheless, our results are compatible with previous studies showing that pure-tone HL is often associated with lower intelligibility without necessarily affecting speech comprehension (Ching and Dillon, 2013; Wingfield et al., 2006). This aspect is particularly interesting as it supports the view that individuals with HL can learn to use cognitive strategies (Heinrich, 2021; Moore et al., 2014) or engage semantic processing (Shao et al., 2022) to compensate for physiologically deficient auditory input and improve speech comprehension. In any case, future studies that allow clarifying the complex relationships between HL, intelligibility and comprehension under different experimental conditions would be helpful to determine the behavioral outcome of different compensation strategies.

Finally, it should be noted that explorative correlation analyses across the entire participant sample yielded a negative association between d-prime scores in the quiet condition (intelligibility) and PTAs. Surprisingly, however, the correlation with the d-prime values of the multi-talker babble noise condition did not reach significance, and a similar non-significant pattern emerged for the relationships between PTAs and speech comprehension. Although this is purely speculative, we point out a phenomenon that occurs frequently in correlational research and is related to the variance of the data. Drawing on this assumption, we may infer that the lower behavioral variability of speech intelligibility in the multi-talker babble condition could be the reason for the lack of relationship between the two variables (Figure 3). Likewise, the non-significant correlations between PTAs and comprehension could in principle also have been influenced by low data variability. Despite this argument, it should be emphasized that speech comprehension can also be influenced by other factors, including syntactic complexity, sentence presentation rate (Wingfield et al., 2006), or the recruitment of additional brain areas outside the canonical speech network to compensate for a poor hearing acuity (Peelle and Wingfield, 2016). Such a perspective would be at least partially supported by previous reports showing that there is no difference in sentence comprehension between normal-hearing and hearing-impaired individuals when processing syntactically simpler sentences, even at fast speech rates (Wingfield et al., 2006).

### 4.4 EEG data

**FIGURE 8:**
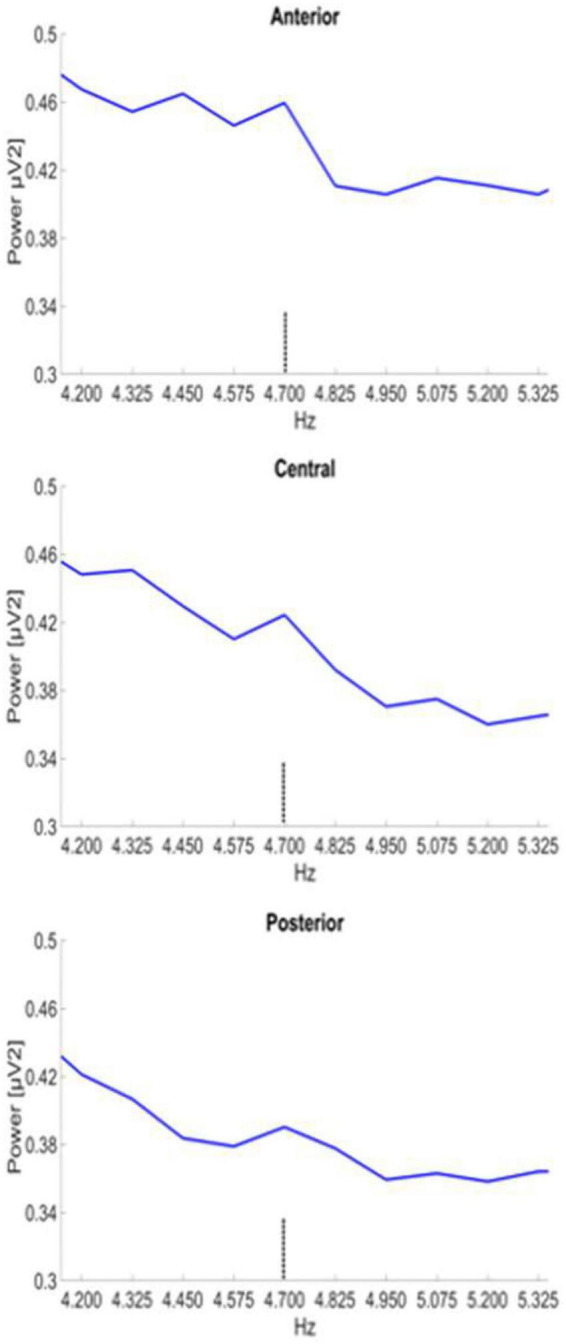
Power spectra in the syllable rate range during the quiet condition are depicted for the CG at anterior (first row), central (second row), and posterior (third row) electrodes. These power spectra are calculated from the EEG signal filtered in the range of 0.5–50 Hz instead of 4–5.6 Hz to show that there is a clear peak in the data corresponding to the average syllable frequency of 4.66 Hz (dashed line).

Although the non-specific anterior-posterior, laterality and anterior-posterior × laterality effects are not relevant to the research questions we addressed, they are still useful for validating the frequency-tagging approach. In fact, these topographic patterns are consistent with previous research showing that the neural representation of syllables is often particularly pronounced at central and midline electrodes (Batterink and Paller, 2019; Buiatti et al., 2009; Rimmele et al., 2023). Furthermore, as shown in Figure 8, in the CG there was a clear peak corresponding to the average syllable frequency of 4.66 Hz (dashed line) in the quiet condition, and since the EEG data included in this figure are filtered between 0.5 and 50 Hz rather than in the 4–5.6 Hz range, they provide a direct link between the EEG measures and syllable processing. This additional data representation increases confidence that modulations in theta-band activity reflected syllable-level processing and cannot be explained solely by non-syllabic aspects of auditory processing or general cognitive engagement. While it is well-established that theta oscillations are involved in speech segmentation and syllable tracking (Meyer, 2018), the present findings extend this framework by demonstrating that theta-band responses encode syllable structure in naturalistic speech under varying levels of acoustic degradation and across different hearing profiles.

Interestingly, the main effects of condition (intelligibility and comprehension) and group (intelligibility) that we identified at the behavioral level were also expressed in the brain parameters. Against this background, the EEG analyses revealed significant interactions between the experimental conditions and the anterior-posterior and laterality factors, and the latter two variables were also modulated by group membership (Figures 4, 5). The condition-related interactions were associated with increased EEG spectral power in the quiet compared to the multi-talker babble noise condition at central and midline electrodes, with maximal expression at sensor Cz. Otherwise, the group×anterior-posterior and group × laterality interaction effects were driven by increased amplitudes in the CG compared to the NTHL sample at anterior and right-sided scalp sites. This constellation of results is particularly interesting for several reasons. Since we restricted the EEG analyses to the syllable domain, the data provide empirical evidence that the neural representation of these specific linguistic units is partially disrupted or at least altered by both background noise and HL. Furthermore, the notion that EEG power spectra were larger in the quiet compared to the multi-talker babble condition with maximum signal strength at electrode Cz can be taken as an indication of down-modulation of auditory sources in the presence of background noise (Ding et al., 2016; Liégeois-Chauvel et al., 1994; Picton et al., 1999; Rimmele et al., 2023). Finally, the generally increased brain responses observed in the CG compared to the NTHL group over anterior and right-hemispheric electrodes may reflect either a neural signature of reduced speech intelligibility associated with hearing impairment in the NTHL group or age-related compensatory recruitment mechanisms in the CG, despite hearing acuity being within the normative range (Anderson et al., 2020; Goossens et al., 2016; Karunathilake et al., 2023). In particular, increased frontal activity has been interpreted in previous work as reflecting greater reliance on domain-general cognitive control and listening effort under conditions of reduced auditory fidelity. Similarly, right-hemispheric contributions have been linked to compensatory processing during challenging or degraded auditory perception in older adults. However, given the cross-sectional nature of the present data, these interpretations remain speculative and cannot be disentangled conclusively.

#### 4.4.1 Effects of condition

The topographical distribution of the increased neural responses we found in the quite compared to the multi-talker babble noise condition is consistent with several previous studies that used different EEG metrics to determine neural syllable representation (Batterink and Paller, 2019; Buiatti et al., 2009; Rimmele et al., 2023). Furthermore, reduced brain activity when listening to speech embedded in noise compared to listening in quiet appears to be an overarching neural principle that has been reported in various EEG and imaging findings, including ERPs (Burkhardt et al., 2022; Dong and Gai, 2021), FFTs (Fiedler et al., 2021), functional connectivity (Baboukani et al., 2021), frequency-following responses (Song et al., 2011), neural speech tracking (Schmitt et al., 2022; Vander Ghinst et al., 2019), temporal response function (Muncke et al., 2022) as well as functional magnetic resonance imaging (Manan et al., 2017).

In principle, altered brain responses in the presence of background noise can be explained by at least three different factors, namely compensatory mechanisms, interference effects and speech acoustics (Campbell and Sharma, 2013; Harmon et al., 2021; Kuruvilla-Mathew et al., 2015). For example, additional recruitment of attention-related brain regions may serve as a compensatory strategy to facilitate auditory stream segregation in a multi-talker babble environment (Paredes-Gallardo et al., 2018; Snyder et al., 2006). On the other hand, listening to speech in background noise can cause linguistic interferences that affect the understanding of spoken language or lead to increased cognitive demands (Harmon et al., 2021). In addition, background noise can alter the spectral and temporal attributes of the target speech signal and thus exert a modulatory influence on brain activity (Hoen et al., 2007). Although there is currently evidence for all of these mechanisms (Campbell and Sharma, 2013; Harmon et al., 2021; Hoen et al., 2007; Kuruvilla-Mathew et al., 2015), the consistently reduced EEG amplitudes we found in the multi-talker babble noise compared to the quiet condition rather favor the hypothesis of interference effects or acoustic influences.

A first argument in favor of speech acoustics is that background noise can disrupt neural synchrony, leading to a degraded speech representation at the cortical level and thus to reduced FFT amplitudes (Anderson et al., 2010). Multi-talker babble noise also limits the detection and discrimination of the target signal through acoustic masking, which increases the spectral and temporal saturation of speech as a function of increasing numbers of background talkers, and results in lower brain activity (Hoen et al., 2007). Despite these acoustic considerations, it is also conceivable that background noise disrupted concentration and attention (Marsh et al., 2015), which was reflected in smaller EEG responses (Alho, 1992; Alho et al., 1994). Finally, there is also the possibility that multi-talker babble noise may have led to linguistic competition because additional background information may have interfered with the target message (Hoen et al., 2007). Certainly, further studies are needed to clearly determine the specific mechanisms underlying the attenuation of EEG responses in a multi-talker babble noise scenario.

#### 4.4.2 Effects of group

Consistent with the purpose of this study, the frequency-tagging analyses brought to light group effects which were reflected in increased EEG responses at anterior and right-sided electrodes in the CG compared to the NTHL cohort. At first glance, these results appear to be in contrast to neural speech tracking studies, which have often found stronger temporal correspondence between the EEG signal and the speech envelope in hearing-impaired individuals compared to normal-hearing participants (Decruy et al., 2020; Fuglsang et al., 2020; Schmitt et al., 2022). However, several linguistic and methodological factors must be considered when interpreting and comparing the results of different studies. The main difference between neural speech tracking and frequency-tagging is that the former relies on time series analysis with the aim of identifying how strongly the brain time-locks to speech acoustics at the sentence level (Zoefel and Kösem, 2024), while frequency-tagging approaches selectively focus on the neural representation of expected low-level linguistic attributes, without considering other influential sources of the signal (Buiatti et al., 2009; Ramos-Escobar et al., 2021). Accordingly, our results do not necessarily contradict previous neural tracking studies, but simply need to be interpreted with a different rationale. Drawing on this background, it is conceivable to believe that the increased neural speech tracking commonly observed in hearing-impaired individuals is simply a manifestation of cognitive strategies to compensate for poor hearing acuity (Schmitt et al., 2022). This viewpoint is at least consistent with correlation analyses that often show a positive relationship between PTA and neural speech tracking measures, with larger associations in individuals with higher levels of pure-tone HL (Decruy et al., 2020; Fuglsang et al., 2020; Schmitt et al., 2022). In contrast, the reduced neural representation of syllables that we found in the NTHL group compared to the CG group based on the frequency-tagging approach could be viewed as a reflection of weakened sensory processing. Nevertheless, such sensory impairment can potentially be overcome at the sentence level through cognitive compensatory mechanisms, as suggested by most of the neural speech tracking studies (Decruy et al., 2020; Fuglsang et al., 2020; Schmitt et al., 2022). This perspective would also be consistent with our behavioral results, which show a disadvantage for hearing-impaired individuals in speech intelligibility, but not in comprehension. Therefore, our results should not be viewed as a contradiction to studies on neural speech tracking, but rather as a useful addition to better understand the holistic effects of HL on speech processing.

Although a comprehensive explanation of the topographic between-group differences we found in the frequency-tagging approach is somewhat difficult, we outline some speculations that can be directly tested by future studies in this research area. Intuitively, the increased EEG parameters we revealed in the CG at anterior and right-sided electrodes could be interpreted as evidence of a pure and more robust syllable representation at the neural level. However, we cannot rule out that these effects were intertwined with cognitive compensatory strategies that may be required to deal with speech comprehension difficulties in older age despite an unremarkable pure-tone hearing threshold (Elmer et al., 2023a; Giroud et al., 2021; Isler et al., 2021; Strouse et al., 1998; Tremblay et al., 2002, 2021). Such an explanation would be supported by previous studies showing that healthy aging may be associated with altered neural representations of syllables or divergent psychometric categorization functions without necessarily having HL (Bidelman et al., 2014; Elmer et al., 2023a; Oron et al., 2019; Strouse et al., 1998; Tremblay et al., 2002, 2021). Hence, it is quite possibly that the individuals of the CG may have mobilized additional cognitive resources at anterior scalp sites and in the right hemisphere to overcome age-related difficulties in speech processing (Anderson et al., 2020; Bartrés-Faz and Arenaza-Urquijo, 2011). Interestingly, the increased EEG power spectra that we observed in the CG at anterior electrodes would also fit into the ‘posterior-anterior shift in aging’ (PASA) model, which postulates that prefrontal regions are over-recruited to preserve cognitive performance due to age-related posterior cortical brain changes (Davis et al., 2008). Furthermore, while right-lateralized activity has been associated with prosodic processing and broader auditory attention mechanisms (Campbell and Sharma, 2013; Giroud et al., 2019; Harmon et al., 2021; Meyer et al., 2003, 2004), evidence from aging and hearing impairment research suggests that sensitivity to prosodic cues may be reduced or altered in older and hearing-impaired listeners (Peelle and Wingfield, 2016), indicating that the present findings do not support a specific interpretation in terms of enhanced lexical stress or prosodic processing and are more cautiously interpreted as reflecting general differences in the distribution of neural resources engaged during speech processing. Finally, it is worth noting that we only tested older participants without measuring an additional sample of young adults. Although our approach is particularly useful for studying the neural correlates of syllable representation while controlling for aging, it has the disadvantage that it cannot properly explain the cognitive operations that gave rise to topographical group differences. In other words, even though we were able to demonstrate a neural weakening of syllable representation in subjects affected by HL, it is not yet clear why the participants whose hearing was in the normative range specifically exhibited increased brain activity at anterior and right-sided electrodes. In this sense, the inclusion of an additional sample of young adults combined with a comprehensive psychometric testing procedure would be helpful to disentangle the effects of neural syllable representation and speech processing strategies.

## 5 Limitations

An important limitation of the present study concerns the composition of the background noise. To rigorously control acoustic and speaker-related features across all conditions, the same speaker was used for both the target language and the background noise. This approach minimized potential confounding factors related to differences in voice identity, pitch, timbre, articulation, and prosody, thus allowing us to more accurately isolate the effects of background noise and listening status on neural frequency-tagging responses. However, this design does not fully replicate the complexity of natural multi-speaker listening situations, where competing speech streams are typically generated by several speakers with distinct voice characteristics. Using different competing speakers could have increased the demands on information masking and required stronger auditory stream separation processes, potentially leading to a greater strain on attentional neural resources. Consequently, our interpretation that compensatory attentional mechanisms can facilitate auditory stream separation in multi-speaker background noise should be considered within the context of the present experimental design. Future studies using more ecologically valid multi-speaker sound conditions will be important to determine how speaker variability affects neural speech processing and auditory stream separation in old age and HL. A further limitation of the present study is that it cannot be conclusively determined whether the observed frequency-tagging responses reflect syllable-level processing per se or are instead primarily driven by lower-level acoustic properties, in particular envelope modulation. Accordingly, a more cautious interpretation is that neural activity in the syllable-rate range likely reflects an interaction between acoustic and linguistic processes. Given that the speech envelope inherently contains periodic structure at syllabic rates, a portion of the observed neural synchronization may be explained by responses to amplitude fluctuations in the acoustic signal. A further methodological consideration is the focus on a narrow frequency band (4–5.6 Hz) as the primary analysis window. This range was selected based on the syllable rate of the stimulus material and was intended to maximize sensitivity to neural activity time-locked to syllabic dynamics while improving signal-to-noise ratio. This approach follows established frequency-tagging studies of continuous speech that target stimulus-defined rhythmic rates. Importantly, this choice does not imply that the observed effects are specific to this frequency range or exclusively reflect syllable-level processing, but rather represents an analysis strategy optimized for the dominant temporal structure of the input. Future work using broader spectral analyses will be important to further evaluate the frequency specificity of the reported effects.

## 6 Conclusion

Using a frequency-tagging approach aimed at exploiting the quasi-periodic syllabic rhythm of sentences presented in quiet or embedded in multi-talker background noise, we extracted syllable representation-specific neural activity in two groups of elderly individuals with and without pure-tone HL. The intelligibility and comprehension indices conjointly confirmed that perceiving speech in noise was more demanding than processing speech in the absence of a disturbance signal. Furthermore, the NTHL group was overall characterized by reduced intelligibility when listening to speech in quiet and background noise. Importantly, the EEG data also clearly demonstrated that both multi-talker babble noise and HL were associated with reduced neural syllable representation, suggesting a common neural principle underlying acoustically and physiologically degraded speech signals. Otherwise, it is important to mention that the absence of significant correlations between the neural frequency-tagging measures and behavioral performance suggests that the observed neural effects may not directly map onto the behavioral indices used in the present study. One possible explanation is that the neural responses indexed here likely reflect relatively early and automatic stages of auditory encoding, whereas the behavioral measures of intelligibility and comprehension depend on additional higher-order processes such as lexical access, decision-making, working memory, and response selection. These later-stage processes may introduce additional variability that obscures direct brain-behavior relationships. Moreover, limited inter-individual variability in behavioral performance, particularly in more favorable listening conditions, may have further reduced sensitivity to detect correlations. Accordingly, while the present neural findings demonstrated modulation by listening condition and hearing status at the group level, their functional significance in relation to overt behavior should be interpreted with caution. Taken together, these results have implications for better understanding how hearing impairment influences the encoding of syllabic information in naturalistic speech and pave the way for future studies aimed at understanding the interactions between HL, speech intelligibility and comprehension.

## Data Availability

The raw data supporting the conclusions of this article will be made available by the authors, without undue reservation.

## Footnotes

1
https://osf.io/s3kzq/
